# Editorial: The emerging discipline of quantitative systems pharmacology

**DOI:** 10.3389/fphar.2015.00129

**Published:** 2015-06-30

**Authors:** Tarek A. Leil, Sergey Ermakov

**Affiliations:** Bristol-Myers Squibb, Clinical Pharmacology and Pharmacometrics/Exploratory Clinical and Translational ResearchPrinceton, NJ, USA

**Keywords:** systems pharmacology, systems biology, pharmacometrics, clinical pharmacology, modeling and simulation, in silico modeling, biomedical research

Quantitative Systems Pharmacology (QSP) has emerged recently as an approach that integrates knowledge coming from multiple disciplines including drug pharmacology, systems biology, physiology, mathematics and biochemistry. QSP was formally defined as a discipline and endorsed in the NIH White Paper (Sorger et al., 2011) in 2011. It has emerged at a time when the pharmaceutical industry is facing growing challenges in efficiency and productivity in R&D. QSP has the potential to help overcome some of these challenges. QSP models allow researchers to evaluate multiple hypotheses in-silico that would otherwise need to be evaluated experimentally. There is an expectation that the use of QSP will reduce the cost of R&D and the risks associated with uncertainties and gaps in our knowledge while bringing new therapies to patients.

QSP models are typically perceived as a research tool for hypothesis generation in drug discovery and exploratory clinical development; however, recently the US FDA used a QSP model to evaluate a proposed drug regimen for a new biologic therapy (Peterson and Riggs, 2015). In their communication with NPS Pharma, the FDA's Clinical Pharmacology division used a published QSP model of the calcium homeostasis system (Peterson and Riggs, 2010) to recommend an alternate dosing regimen for NATPARA, an injectable parathyroid hormone replacement drug used to control low blood calcium in patients with hypoparathyroidism. The use of a QSP model by the FDA to recommend an alternate dosing regimen to a sponsor highlights one of the important future applications of QSP models in regulatory interactions, and also represents an important milestone for the field. It is the first public instance of a QSP model being used by a regulatory agency to make a clinical recommendation to a sponsor. In the future, it is anticipated that it will be sponsors that leverage the utility of QSP models to support their own clinical decision making with regulatory agencies.

In the present research topic entitled, “The Emerging Discipline of Quantitative Systems Pharmacology,” we provide an introduction to the developing field of QSP with a series of articles that describe models in different disease areas; showing how these models can be used to evaluate important research questions in pharmaceutical R&D. The research topic starts with a perspective article by Leil and Bertz (2014) that describes the history of how modeling tools where used in pharmacology and in drug development. The authors point out two major reasons for the growing importance of the QSP approach, (i) difficulty in finding new targets for therapies, and (ii) increasing cost and time required for developing a successful drug. Integrated into the drug development process, QSP modeling could become an effective tool to facilitate R&D; for example to translate knowledge between experimental systems (e.g., animal to human), and to predict the effects of multiple therapeutic interventions in combination; a task that would be inefficient using only clinical experimentation. The other articles in the research topic go on to demonstrate applications of QSP models to influence decision making in biomedical research.

One of the important applications of QSP modeling in pharmaceutical R&D is optimization of clinical dose and schedule. Oncology is one of the disease areas where the narrow therapeutic window of most therapeutics demands fine tuning of dose and schedule. Utilization of high doses of anti-angiogenesis therapy can result in rapid suppression of angiogenesis and hypoxia leading to tumor shrinkage. Paradoxically, this can subsequently lead to reduced drug delivery to the tumor and resumption in tumor angiogenesis, followed by progression of tumor growth. The paper by Sharan and Woo (2015) discusses how to delay or prevent this from happening by optimizing the dose and regimen of the anti-angiogenesis therapies with a QSP model of angiogenesis.

Another important application of QSP models is to provide mechanistic explanations for clinical data that are often counterintuitive to the perceived mechanism of action (MoA) of a drug. Despite the fact that two SGLT2 inhibitors are already approved for use in patients, questions remain regarding their MoA and why efficacy is lower than expected based on their high potency and selectivity for SGLT2. The papers by Demin et al. (2014) and Lu et al. (2014) explored this issue using mechanistic models of renal tubular filtration and transport, incorporating the PK and MoA of SGLT2 inhibitors. Lu et al. (2014) proposed two possible explanations for the lower than expected efficacy; the residual activity of SGLT2 following inhibition in the renal tubules, and the compensatory effect of SGLT1. Demin et al. (2014) supported this hypothesis, but also offered an alternative in which the sites of action of SGLT2 inhibitors are located not in the lumen of the kidney's proximal tubules where the concentration of SGLT2 inhibitor is high, but perhaps in the proximal tubule where the concentration of inhibitor is lower. Complex dose-response dependencies are often encountered in many disease areas, for instance, in treatments of schizophrenia as investigated by Spiros et al (Spiros et al., 2014). With the use of a sophisticated QSP model of cognitive impairment in schizophrenia, the authors predicted an inverse U-shape dose-response with glycine that is a consequence of the shifting balance between excitation and inhibition in the cortical network.

The application of mechanistic models for prediction of target dependent or independent toxicity in secondary tissues has been the focus of QSP for many years, as this is the most common reason for termination of the development of otherwise efficacious therapies. In order to predict toxicity using a mechanistic model, it is useful to incorporate a physiologically based PK (PBPK) model to predict drug concentrations in the target organ. Woodhead et al. (2014) described the use of a PBPK/toxicity model of drug induced liver injury (DILI) to evaluate the impact of bile salt export pump (BSEP) inhibition on hepatotoxicity in rats and humans. The DILI model was used to predict the responses to BSEP inhibitors with and without clinical hepatotoxicity. In accordance with the observed clinical data, the model predicted that bosentan, but not telmisartan, will cause mild hepatocellular ATP decline and serum ALT elevation. Similar to the research by Woodhead et al. (2014), Chetty et al. also relied on PBPK to predict drug concentrations in tissue, linking these concentrations to target-mediated pharmacodynamic (PD) effects (Chetty et al., 2014). They did so using the Simcyp PBPK simulator, a tool that has a built-in PBPK model and allows users to input drug specific parameters that have been measured *in-vitro* to predict *in-vivo* plasma and tissue PK. Simcyp has become an important tool for pharmaceutical R&D and for communicating with regulatory agencies regarding the PK of investigational drugs and their potential PK-related drug-interactions. Chetty et al. described the use of Simcyp to predict the tissue concentrations of four different drugs, metoprolol, nifedipine, triazolam, and zolpidem (Chetty et al., 2014). They demonstrated how polymorphisms in drug metabolizing enzymes would have an effect on the concentration of drugs in the target tissue and the subsequent impact on pharmacodynamics.

One of the technical issues that potentially limit more widespread use of QSP in biomedical research is the lack of an accepted standard modeling tool to facilitate sharing of models between researchers. The tool should permit evaluation of experimental scenarios of interest in a flexible computational environment for conducting efficient high throughput simulation. A potential solution was implemented in the web-based virtual systems pharmacology (ViSP) platform described in the article by Ermakov et al. (2014). The salient feature of ViSP is the use of a model in the form of an executable file. Matched with a full set of model parameters this executable becomes independent of the model structure and the software that were used to develop the model while preserving flexibility in the input parameters. These characteristics could be useful in the future when the utilization and sharing of QSP models becomes more widespread.

In conclusion, we would like to emphasize the emerging potential that QSP holds for biomedical research and in particular to improve decision making in pharmaceutical R&D. In order for this to occur, QSP will need to present more examples of the value that it can bring to the process of hypothesis generation and testing; examples like those included in this research topic.

## Conflict of interest statement

Both of the authors are employees of Bristol-Myers Squibb. The authors declare that the research was conducted in the absence of any commercial or financial relationships that could be construed as a potential conflict of interest.
